# Investigating errors in medical imaging: medical malpractice cases in Finland

**DOI:** 10.1186/s13244-021-01011-8

**Published:** 2021-06-28

**Authors:** Tarja Tarkiainen, Miia Turpeinen, Marianne Haapea, Esa Liukkonen, Jaakko Niinimäki

**Affiliations:** 1 Department of Diagnostic Radiology, Research Unit of Medical Imaging, Physics and Technology, Oulu University Hospital, Oulu, Finland; 2 Administrative Centre, Research Unit of Biomedicine, Oulu University Hospital and University of Oulu, Oulu, Finland; 3 Department of Diagnostic Radiology, Medical Research Center, Oulu University Hospital and University of Oulu, Oulu, Finland; 4 Department of Diagnostic Radiology, Oulu University Hospital, Oulu, Finland; 5 Department of Diagnostic Radiology, Research Unit of Medical Imaging, Physics and Technology, Oulu University Hospital and University of Oulu, Oulu, Finland

**Keywords:** Safety error, Incident reporting, Adverse events, Patient injury claim

## Abstract

**Objective:**

The objectives of the study were to survey patient injury claims concerning medical imaging in Finland in 1991–2017, and to investigate the nature of the incidents, the number of claims, the reasons for the claims, and the decisions made concerning the claims.

**Materials and methods:**

The research material consisted of patient claims concerning imaging, sent to the Finnish Patient Insurance Centre (PVK). The data contained information on injury dates, the examination code, the decision code, the description of the injury, and the medical grounds for decisions.

**Results:**

The number of claims included in the study was 1054, and the average number per year was 87. The most common cause was delayed diagnosis (404 claims, 38.3%). Most of the claims concerned mammography (314, 29.8%), radiography (170, 16.1%), and MRI (162, 15.4%). According to the decisions made by the PVK, there were no delays in 54.6% of the examinations for which claims were made. About 30% of all patient claims received compensation, the most typical reason being medical malpractice (27.7%), followed by excessive injuries and injuries caused by infections, accidents and equipment (2.7%).

**Conclusion:**

Patient injury in imaging examinations and interventions cannot be completely prevented. However, injury data are an important source of information for health care. By analysing claims, we can prevent harm, increase the quality of care, and improve patient safety in medical imaging.

## Key points

- Adverse events in medical imaging result in patient injury and lead to compensation claims.
- The majority of claims concern delayed diagnosis.
- Collecting and analysing patient injury claims is important for increasing and improving patient safety.

## Introduction

Medical imaging with diagnostic interventions is significant not only for diagnosing patients’ diseases, but also for treating cancer [1]. According to international studies, the incidence of adverse events among hospital patients varies between 4 and 17% of all patients, and 30%–75% of these are preventable. In radiology, the incidence of injuries is about 1%, and 35% of these are preventable [2–4]. These adverse events can lead to inaccurate or delayed diagnoses as well as unsuccessful treatments or physical injury to a patient [5–7]. A patient has the right to expect beneficial and safe examination or treatment. It is important that in medical malpractice cases, a patient can request an explanation for any failure and claim for compensation.

The Finnish statutory patients’ insurance system is based on the Finnish Patient Injuries Act (585/1986), 1 May 1987. This Act safeguards the rights of both patients and health care personnel. In accordance with the law, a patient has the right to apply for compensation if they have suffered a physical injury through medical treatment or health care [8]. In 1986, the Act was globally unique, and only Sweden had a voluntary patient insurance scheme, established in 1975 [9]. At present, all Nordic countries have a similar no-fault (no-blame) scheme, based on the principle of avoidability [8]. The tort liability system, based on patients being compensated if it can be proved that the harm was due to negligence, is different to the no-fault system [10]. The tort liability system is in use in, for example, the United States, the United Kingdom and Italy [11–14].

The Finnish Patient Insurance Centre (PVK) manages and resolves all notifications of patient injuries that have occurred during medical treatment and health care in both the public and private sector. The PVK also promotes patient safety by conducting research, making calculations and compiling statistics [15]. All Finnish health care operators have access to these statistics.

Previous studies of medicolegal claims concerning medical imaging have focused on diagnostic errors such as delayed diagnosis [5], misdiagnosis [16], and the role of the radiologist [6, 17–19]. In the United States, nearly 60% of coded medical malpractice claims are diagnosis related. Almost 50% of all the claims covered computer tomography (CT) (20%), mammography (11%), magnetic resonance imaging (MRI) (10%), and ultrasound (4%) [18]. Errors in medical imaging were categorised as diagnostic or other errors [7, 19, 20]. Diagnostic errors included incorrect, delayed and missed diagnoses, and other errors were, for example, communication errors or equipment and system failures [17, 19]. We know that errors are both inevitable and avoidable, but awareness of potential biases and precise attention to the processes and system issues leading to mistakes and the use of appropriate strategies can reduce the effects of mistakes [21].

Our aim was to investigate the adverse events leading to patients’ claims, and to make health care professionals more aware of them in order to improve patient safety. More specifically, our aim was to investigate what kinds of adverse events occurred in the various imaging modalities and to determine their consequences.

## Materials and methods

### Study design

The PVK granted permission for this retrospective study in July 2018. A de-identified database of patient incident reports was sent digitally by authority of the PVK to the researcher. To protect anonymity, the de-identification covered the patients, radiologists, and other health care personnel on whom the patient’s claims focused. The names of the Finnish regional and university hospitals, private institutions and other identification data of the radiological units were also removed.

### Data selection

We collected data on the claims from 1991 to 2017 and included claims concerning radiography, CT, MRI, ultrasound, fluoroscopy, and interventional and vascular radiology modalities. Nuclear medicine, radiotherapy, cardiology, gynaecology, and ultrasound related to pregnancy and foetus monitoring were excluded. Cardiac and pregnancy examinations were only included if they were related to conventional imaging, for example, radiography, CT or MRI.

### Data analysis

The data were analysed using IBM SPSS Statistics for Windows version 25 (IBM Inc.2017). The excel (Microsoft Excel 2016) data obtained from the PVK contained information on the year and date of the decision, the date of the injury, processing ID, the code and date of the decision, the specialisation, the code of the examination (e.g., AA1AD = head CT), the patient’s underlying disease, and a description of the event. From these data, we extracted information on the radiology modalities, subspecialities, patient’s incurred injury, estimated delays in diagnosis, decisions, and compensations. The number of claims against modalities and the patients’ medical grounds for their claims (delayed diagnosis or medical treatment, incorrect or inadequate diagnosis / examination, accident, infection or complication), were calculated as percentages of the data. In addition, the PVK’s decisions; medical or minor malpractice, unavoidable or excessive, no correlation, injury by equipment, accident or infection were also calculated. The PVK’s decisions are based on criteria defined in the Patient Act: treatment/infection/accidental/equipment-related and unreasonable injuries, injury arising from damage to premises or treating equipment and injury due to incorrect delivery of pharmaceuticals [8] and whether or not the patients have received compensation. In addition, patient injury types were divided into eight different categories (no major harm or no correction to medical care; unavoidable complication or always potential consequences; pain, but no effect on treatment or prognosis; degenerated prognosis or disease or delayed cancer treatment; extra/unnecessary/delayed or inadequate treatment or intervention/deterioration of disease; aches and pains; complication/infection/reinfection/accident or harm; and death or decreased life expectancy).

## Results

### Extent of patient injury claims

**Fig. 1:**
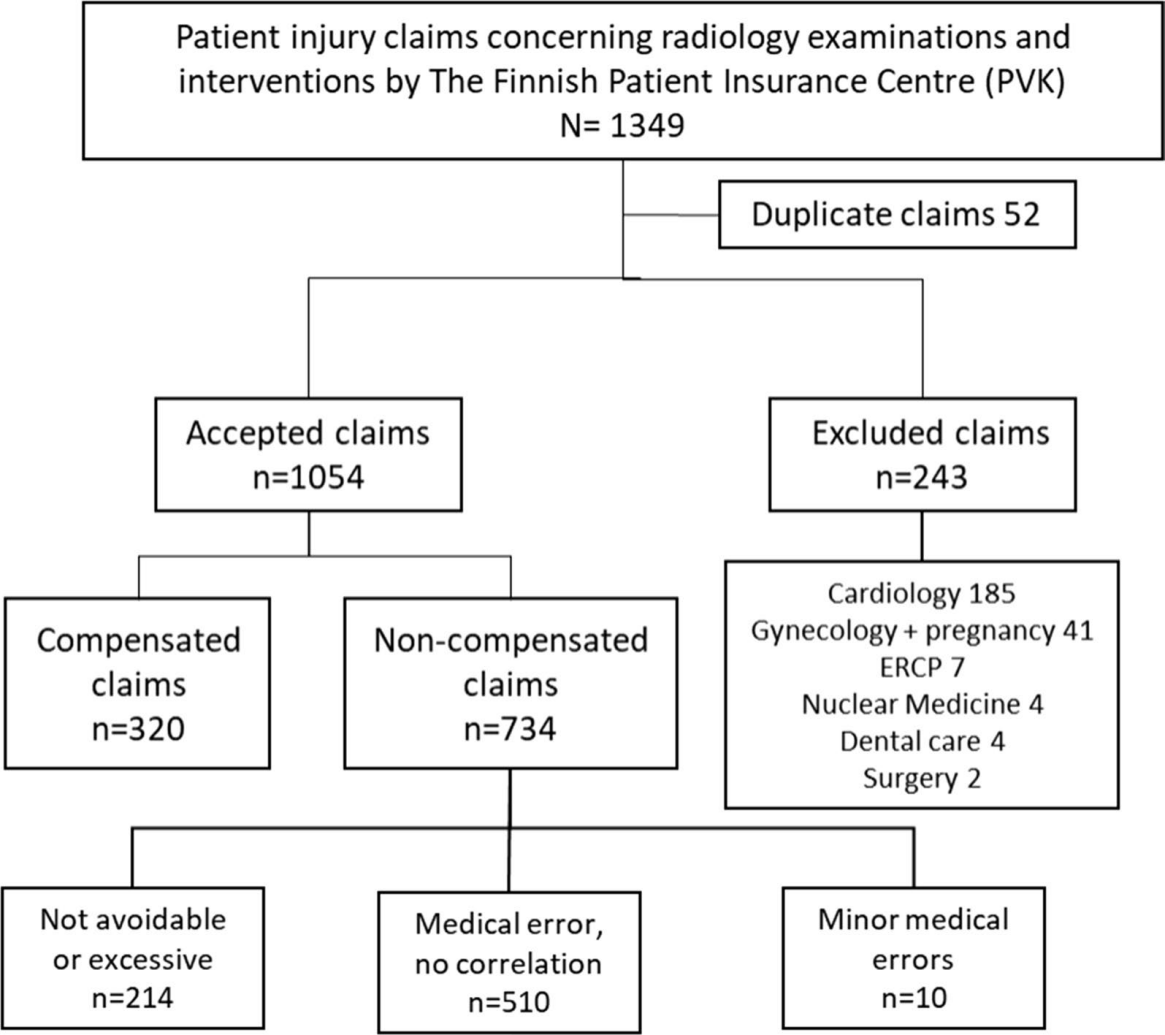
Flowchart of patient injury data collection

**Fig. 2:**
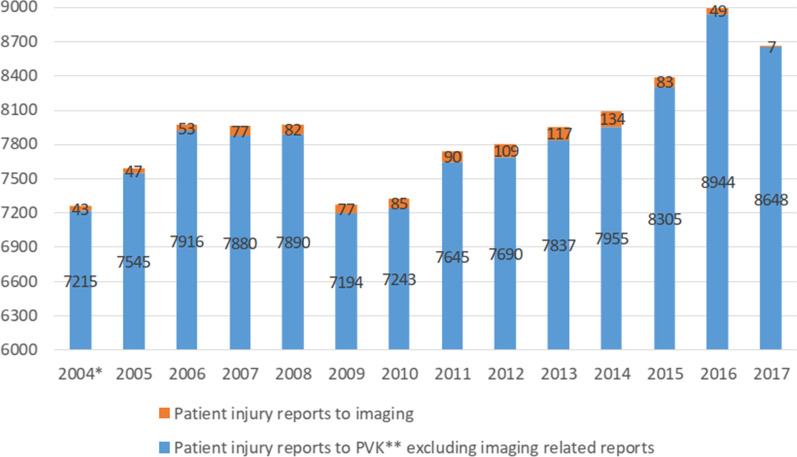
Number of patient injury reports in 1991–2017 in Finland

The exclusion of 243 claims is explained in the data section paragraph above (Fig. 1), and the final included data consisted of 1054 claims. The cases were resolved at the PVK between 1991 and 2017. Figure 2 shows the annual number of patient claims concerning imaging.

During 1991–2004, a total of 43 injury reports related to imaging were registered; thus, only a few per year. After 2005, the number of medical imaging claims has been more than 50 per year. The highest number of claims was recorded in 2014, at 134. The number of claims from 2015 to 2017 was lower but this is not necessarily final, as patients have three years to file a complaint. The average number of patient injury reports concerning imaging has been about 87 reports per year (from 2005 to 2014). The number of patient claims related to medical imaging has increased annually after 2009, as have the number of all claims reported to the PVK.

### Reasons for claims

Delayed diagnosis was the most common cause (38.3%) of patient claims (Table 1). Medical malpractice, infection or complication was second (33.7%) and incorrect or inadequate diagnosis was third (16.8%). The majority of the claims that were related to infections and complications concerned diagnostic and therapeutic vascular and interventional radiology (64.2%). However, when all the modalities are taken into account, the majority of all claims were related to mammography and screening mammography (29.8%), radiography (16.1%) and MRI (15.4%).

**Table 1:** Patient’s reasons for claims in different radiology modalities

Patient’s reasons for the claims	Delayed diagnosis		Incorrect or inadequate diagnosis		Incorrect or inadequate examination		Medical malpractice, accident		Medical malpractice, infection or complication		Delayed medical treatment		Total	
	*n*	%	*n*	%	*n*	%	*n*	%	*n*	%	*n*	%	*n*	%
Radiography	85	50.0	56	32.9	4	2.4	7	4.1	13	7.6	5	2.9	170	16.1
Computed tomography	27	26.2	18	17.5	8	7.8	3	2.9	38	36.9	9	8.7	103	9.8
Imaging	42	25.9	51	31.5	12	7.4	6	3.7	42	25.9	9	5.5	162	15.4
Ultrasound	27	43.5	15	24.2	11	17.7	0	0.0	8	12.9	1	1.6	62	5.9
Interventional radiology^a^	1	1.0	2	2.0	2	2.0	2	2.0	91	91.9	1	1.0	99	9.4
Mammography and screening mammography	218	69.4	35	11	27	8.6	7	2.2	26	8.3	1	0.3	314	29.8
Diagnostic and therapeutic vascular radiology	4	2.8	0	0.0	0	0.0	2	1.4	137	95.1	1	0.7	144	13.6
Total	404	38.3	177	16.8	64	6	27	2.6	355	33.7	27	2.6	1054	100

^a^Biliary intervention, catheter placement, genitourinary, pain management, contrast-enhanced and fluoroscopy examinations

The patients had themselves estimated the time taken for the delayed diagnosis. In its decision, the PVK verified and assessed these delays, as presented in Table 2. The different anatomical regions of the delays were also calculated. Of the examinations, 54.6% had no delays, and of the claims, 16.4% delays were unknown or not mentioned. Most of the long delays (over one month) were in mammography (10.4%) and skeletal examinations (5.6%). More than half of the delays were related to either breast (30%) or skeleton examinations (30%), followed by diagnostic and therapeutic vascular radiology (14%) Fig. 2.

**Table 2:** Diagnosis delay by studied anatomical organs assessed by PVKª

Delay	Brain		Thorax		Breast		Abdomen		Skeleton		Vascular system		Others^b^		Total	
	*n*	%	*n*	%	*n*	%	*n*	%	*n*	%	*n*	%	*n*	%	*n*	%
No delay	39	6.8	21	3.7	155	27	53	9.2	138	24	138	24	31	5.4	575	54.6
1–7 days	2	3.9	5	9.8	7	13.7	13	25.3	16	31.4	2	3.9	6	11.8	51	4.8
Under 2 weeks	4	44.4	0	0	0	0	0	0	4	44.4	0	0	1	11.1	9	0.9
Under 1 month	2	16.7	1	8.3	0	0	0	0	2	16.7	0	0	1	8.3	12	1.1
1–3 months	8	18.6	2	4.7	10	23.3	1	2.3	21	48.8	0	0	1	2.3	43	4.1
4–6 months	4	6.9	4	6.8	27	46.6	3	5.2	18	31	0	0	2	3.4	58	5.5
7–12 months	2	6.9	2	6.9	21	72.4	2	6.9	2	6.9	0	0	0	0	29	2.6
One to 3 years	13	14.8	5	5.7	50	56.8	5	5.7	14	15.9	0	0	1	1.1	88	8.3
4–5 years	2	28.6	0	0	2	28.6	1	14.3	2	28.6	0	0	0	0	7	0.7
Over 5 years	5	55.6	0	0	0	0	2	22.2	2	22.2	0	0	0	0	9	0.9
Delay not known or mentioned	1	0.6	3	1.7	45	26	14	8.1	91	52.6	13	7.5	6	3.5	173	16.4
Total	82	7.8	43	4.1	317	30.1	94	8.9	316	30.0	153	14.5	49	4.6	1054	100

^a^The Finnish Patient Insurance Centre

^b^Thyroid, muscles

### Compensation for claims

**Fig. 3:**
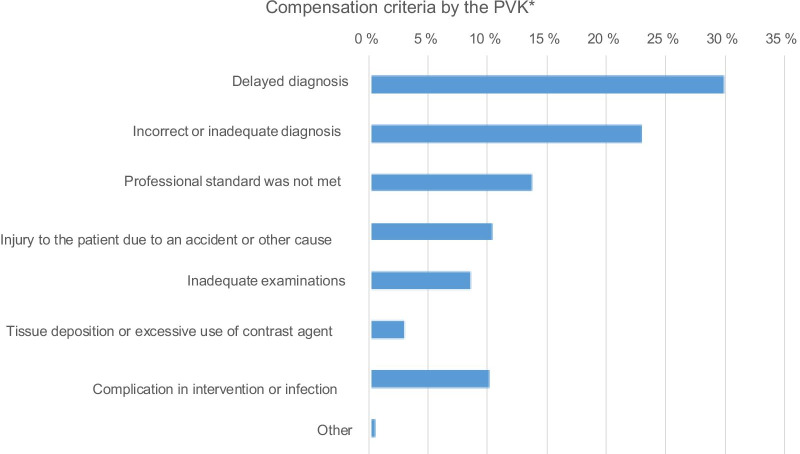
Medical grounds for compensation criteria by PVK

Of all the reported medical imaging-related claims, the PVK found no medical grounds for compensation in 70% of cases, 45% of which had no association with injury, 24% were unavoidable or their consequences were not excessive, and 1% was due to other reasons. Thirty percent of the claims led to patients receiving compensation. Figure 3 presents the medical grounds for the PVK’s decisions. The three most common reasons for compensation were delayed diagnosis (30%), incorrect or inadequate diagnosis (23.1%), and professional standards not being met (13.6%). Accidental injuries and complications or infections accounted for 20% of compensations.

Table 3 shows how compensated and non-compensated decisions were divided among radiology subspecialties. Patients received compensation for medical malpractice (27.7%) or excessive injuries and injuries caused by infections, accidents and equipment (2.7%). Excessive injuries were associated with reduced life expectancy or death. Regarding all subspecialties, the most common reasons for claims were related to musculoskeletal (30.5%) and breast (30.1%) imaging. Decisions that saw ‘no correlation with medical injury’ most common concerned breast imaging (67.5%), followed by musculoskeletal imaging (44.2%). According to the compensation decisions, the most excessive or unavoidable injury cases occurred in vascular interventional imaging (60.4%).

**Table 3:** Distribution of criteria for compensated (medical malpractice and equipment, infection, accident or excessive injury) and non-compensated decisions in radiology subspecialties

Radiology subspecialty	Medical malpractice		Minor medical malpractice		Not avoidable nor excessive		Medical care, no correlation		Equipment, infection, accident or excessive injury		Other, no correlation		Total	
	*n*	%	*n*	%	*n*	%	*n*	%	*n*	%	*n*	%	*n*	%
Neuroradiology	39	49.4	2	2.5	6	7.6	31	39.2	1	1.3	0	0	79	7.5
Thoracic imaging	22	32.4	2	2.9	16	23.5	22	32.4	3	4.4	3	4.4	68	6.5
Breast imaging	61	19.2	1	0.3	30	9.5	214	67.5	3	0.9	8	2.5	317	30.1
Abdominal imaging	26	23.6	1	0.9	34	30.9	34	30.9	3	2.7	12	10.9	110	10.4
Musculoskeletal imaging	131	40.8	3	0.9	32	10.0	142	44.2	8	2.5	5	1.6	321	30.5
Vascular interventional imaging	13	8.2	1	0.6	96	60.4	30	18.9	10	6.3	9	5.7	159	15.0
Total	292	27.7	10	0.9	214	20.3	473	44.9	28	2.7	37	3.5	1054	100

**Fig. 4:**
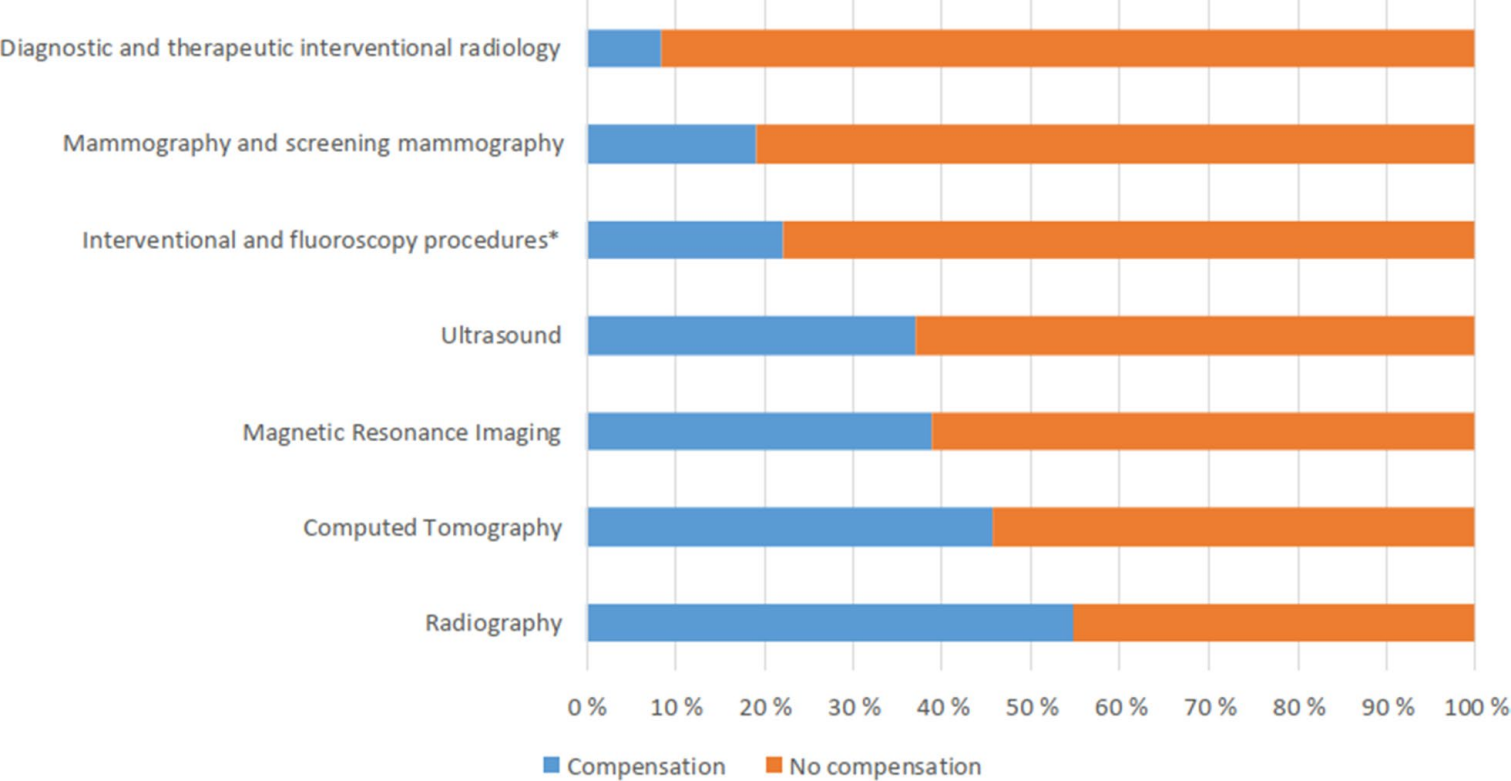
Comparison of compensation payments related to different imaging modalities

Compensations and non-compensations differed between the modalities (Fig. 4). Compensation was most often paid for radiography (54.7%) and CT (45.9%). Almost 40% of reimbursed complaints were related to MRI (38.9%) and ultrasound (37.1%). Compensation was infrequently paid for diagnostic and therapeutic interventional radiology (8.3%), mammography/screening mammography (19.1%) and interventional or fluoroscopy procedures (22.2%).

Information on patients’ injury types were collected from narrative reports (Table 4). ‘Unavoidable or always a potential consequence’ was typical for interventional radiology (75.3%), as well as ‘Complication, infection or other harm’ (34.8%). ‘Pain but no effect on treatment or prognosis’ (69.2%) and ‘Deteriorated prognosis or delayed cancer treatment’ were general in mammography screening (66.7%), and ‘Aches and pains’ were typical in radiography (63.2%). ‘No major harm, or no correlation with medical care’ was most commonly associated with all modalities (38%).

**Table 4:** Categories of patient injury types related to different modalities

Patient injury type	Radiography		Computer tomography, CT		Magnetic Resonance Imaging, MRI		Ultrasound		Interventional radiology^a^		Mammography and screening mammography		Total	
	*n*	%	*n*	%	*n*	%	*n*	%	*n*	%	*n*	%	*n*	%
No major harm, no correlation to medical care	61	15.3	28	7	93	23.3	35	8.6	50	12.5	133	33.3	400	38
Unavoidable complication or always potential consequence	2	1.1	18	9.7	2	1.1	9	4.8	140	75.3	15	8.1	186	17.6
Pain, but no effect on treatment or prognosis	16	10.1	14	8.8	12	7.5	5	3.1	2	1.3	110	69.2	159	15.2
Degenerated prognosis or disease or delayed cancer treatment	3	10	1	3.3	3	10	3	10	0	0	20	66.7	30	2.8
Extra, unnecessary, delayed or inadequate treatment or intervention, deterioration of disease	32	28.6	17	15.2	23	20.5	9	8.0	3	2.7	28	25	112	10.6
Aches and pains	48	63.2	4	5.3	13	17.1	8	10.5	1	1.3	2	2.6	76	7.2
Complication, infection, recompiled infection	5	7.2	17	24.6	12	17.4	7	10.1	24	34.8	4	5.8	69	6.5
Death or decrease life expectancy	1	4.5	5	22.7	6	27.3	1	4.5	9	40.9	0	0	22	2.1
Total	168	15.9	104	9.9	164	15.6	77	7.3	229	21.7	312	29.6	1054	100

^a^Diagnostic interventional and vascular radiology and fluoroscopy examinations

## Discussion

In Finland, 714 radiology examinations were performed per thousand inhabitants in 2015 (approximately 3.9 million examinations in a 5 million population). The proportion of native/contrast X-ray examinations was approximately 86%, CT was 11%, X-ray or CT-guided procedures 1%, angiographies 1%, and CTD examinations 1%. In addition, 640,000 ultrasounds and 390,000 MRI examinations were reported [22].

In Finland, patients file imaging-related complaints every year. The number of patient injury claims started to increase after 2005 and the number of annually filed complaints almost doubled. One possible reason for this is the change in patient safety culture. In 1999, the Institute of Medicine (IOM) published an article called ‘To err is human’ [23]. This pioneering research also increased the number of patient safety studies in Finland and increased public awareness of patient safety issues [24]. The number of patients’ injury claims increased by 20% between 2010 and 2016. This trend is consistent with the general development in Nordic countries [25] and Australia [26], although in the United Kingdom, claims have only increased by 9.3% [27]. In the United States, radiology is the eighth most likely service to be implicated in medical malpractice claims [28]. The risk of litigation for radiologists has also increased in Italy, and it is estimated that 44% of Italian radiologists will receive a malpractice claim over a period of 10 years. [13].

Because this study is the first to analyse patients’ injury claims concerning medical imaging in Finland, we wanted to collect data from as long a period as possible to obtain a broad view of the claims. The acquired data cover all medical imaging claims in both public and private health care. A notification of injury must be filed within three years of the date on which the patient was first informed of their injury [25]. There was a decrease in notification volumes from 2009 to 2010 (Fig. 1). According to PVK’s research (personal communication November 2019) the most feasible explanation for this was the financial crisis during these years. In various social crises, patient injury-reporting activity first decreases, but later begins to increase. A similar situation has emerged during the COVID-19 pandemic (May 25, 2020). This statement is based on a general observation and has no actual scientific evidence.

In 2013, imaging accounted for about 1.5% (117/7954) of all patient complaints to the PVK, and in 2014, 1.7% (134/8089) [25]. Although imaging accounts for less than 2% of all patient claims, it should be noted that not all patient injuries are reported to the Patient Injury Centre. In addition, health care personnel report patient injuries and adverse events to several authorities, including the Radiation and Nuclear Safety Authority (STUK), the National Supervisory Authority for Welfare and Health (VALVIRA) and the Reporting System for Safety Incidents (HaiPro). If we compare the annual reports made by patients with those made to STUK by imaging staff (293 reports per year), we see that health professionals report adverse events three times more often than patients [29].

According to several previous studies, most claims have concerned mammography, radiography, and CT examinations [16, 18, 20, 23]. In Finland also, the highest number of patients claims have concerned mammography (22.8%), the second highest radiography (15.9%), the third highest MRI (15.6%) and the fourth highest CT (9.9%). According to our results, the reason why MRI has overtaken CT in the number of related complaints is that patients make more claims concerning delayed or incorrect diagnoses related to MRI than to CT. This in turn is due to musculoskeletal imaging being more frequent, with MRI making up 66% of all MRI-related claims. For the same reasons, the PVK has paid more compensation (19.7%) for MRI-related claims than for those related to CT (14.7%). It is notable that as many claims of delayed diagnosis (27 claims) are related to ultrasound as to CT. The PVK has paid six patients compensations related to ultrasound procedures. The reasons were for instance, ‘misinterpretation of the ultrasound, no gallstone, unnecessary gallbladder removal’ and ‘biceps muscle rupture was not discovered in ultrasound, found in MRI, diagnosis delayed by three months’.

Among radiology specialties, the claims most commonly concerned musculoskeletal and breast imaging. Similar results have been observed in other countries and by other studies [13, 14, 17, 18, 24]. The reasons for medical malpractice claims against radiologists have been studied widely [13, 14, 24, 30–32]. The most common reasons for these claims are related to diagnostic errors, faulty visual reasoning, cognitive reasoning, pattern recognition, or delayed diagnosis. According to other studies, 10%–30% of breast cancers are missed in mammography [13, 16, 17, 20, 33], other misses are related to bone fractures [11, 13, 33] and lung cancer [31, 34]. In our study, the most common reason for a claim was delayed diagnosis after mammography (54%), radiography (21%) and MRI (10%), as shown in Table 2.

According to the PVK’s decisions, 29% of all compensated claims concern delayed diagnosis. The length of the delay varies from one day to over five years. In acute settings, even a day's delay in diagnosis can be fatal, whereas in other cases, the patient's condition may not deteriorate over time. The following PVK decisions concerning mammography procedures are examples of delays; ‘Breast cancer, mammogram, diagnosis delay six months. No effect on treatment or prognosis, not reimbursable’, and ‘Mammography images interpreted as benign, a finding of malignancy would have required further investigation. Diagnosis delay of breast cancer about two years. Reimbursable’. In the patient claims, the longest delays (1–5 years) were often associated with cancers. Some cancers (e.g., breast, lung, abdomen, muscular) were not observed until several years later and the patients’ claims concerned misinterpretation. It is noteworthy that although late diagnosis may not affect a patient's treatment strategy, it may worsen a cancer patient's prognosis [9].

According to the PVK, in 13.6% of the reimbursed cases, the interpretation of the study was incomplete or incorrect and did not meet the professional standard. Examples of compensable medical malpractices are: *‘*Professional standards were not met in the interpretation of CT scans and the diagnosis and treatment of cerebral infarction was delayed by about a month. If a stroke had been diagnosed a month earlier, appropriate medication could have been started and partial (not complete) deterioration in condition would have been avoided’ and ‘The radiologist should have noticed the abnormality of the patient’s CT scan and performed an emergency MRI. Diagnosis of cerebral abscess was delayed by about two days’. Overall, professional standards were not met in 7.9% of all claims.

Out of the 22 (2.1%) fatal or life expectancy-shortening cases, nine were related to interventional radiology, six to MRI, five to CT, and one to radiography and ultrasound. For instance, in the following cases, delay in diagnosis and treatment contributed to symptoms and shortened the patient’s life expectancy; ‘There was a delay in the treatment of recurrent aneurysm and the patient died before treatment’, ‘Bleeding after a thin needle biopsy caused airway obstruction leading to death’, and ‘A brain tumour could have been diagnosed earlier from MRI images’.

Comparison of public and private health care in Finland shows that, for example, in 2017, 54% of all paid patient injuries were reimbursed in the public sector and 46% in the private sector. In 2014, a total of EUR 39.9 million were compensated, of which medical imaging accounted for 1.7% (EUR 67,830). The majority (91.2% in 2017) of compensable injuries were classified as treatment injuries. The reimbursement criteria were based on the fact that an experienced health care professional would have acted differently and avoided the injury [18]. For example, ‘During the X-ray examination, the patient fell. The patient’s condition had deteriorated, which should have been observed. Supervision did not meet the required level of professional competence. Caused lumbar fracture. Compensable damage’. In Finland, compensation is not paid for minor medical malpractice. An injury is considered minor if it only causes slight pain, no permanent functional disability, no aesthetic injury, or if the costs incurred do not exceed EUR 200. Minor personal injuries do not require hospital treatment, improve in one to two weeks without any harm, or cause incapacity for work for up to 2 weeks. In 2011, the compensation for a mild and temporary injury was between EUR 200 and EUR 1000, and for a permanent injury of medium severity between EUR 23,100 and EUR 26,400 [8, 25, 35, 36].

Patients claim compensation by filing a notice of injury (an electronic note or pdf form) within three years of the date injured occurred [8]. The notice of injury is registered, and the information and reports needed are acquired from a health care provider and medical expert. Health care professionals have the opportunity to give their statement. The patient is also heard when necessary. After the requested documentation is received, the case is assessed from a medical point of view. The PVK’s medical adviser makes a statement and the patient receives a written claims decision within 7.5 months. The amount of compensation is determined by the guidelines issued by the Traffic and Patient Accident Board [8, 25, 36].

In Finland, approximately 30% of patients' medical injury claims are reimbursed [30]. This rate is quite low in comparison to Sweden, at 49.5% [37] and the United States, where reimbursement rates vary between 40 and 56% [38, 39]. One reason for these low rates in Finland is that the law requires a direct connection between the failure of the duty and the harm caused [Patient Act]. In medicine, it is often difficult to distinguish which adverse events and errors are due to the examination itself and which are due to the related treatment [33]. According to our data, 45% of patient claims are associated with medical care and have no correlation with injury, and in 24% of cases, the damage could not have been avoided or was not excessive.

Our study has limitations. The patient claims analysed were from a single country. However, we believe that problems are similar in other countries. Some of the claims were missing because the three-year appeal period had not yet expired, and therefore the data from 2015 and 2017 were most likely incomplete. Some of the research data were collected from interpretive narratives and may lacked information. In addition, because the data were collected through a computer search and not directly by researchers, they may have contained errors or omissions.

Unfortunately, imaging can cause adverse events that affect patients. Although most of these are minor, some are serious, even fatal. Delayed and incorrect diagnoses cause patients additional and unnecessary pain and uncertainty. It is likely that only a proportion of patient claims are reported to the authorities. Due to this, and in order to improve imaging safety, processing and analysing patient claims is essential. This study focused primarily on patients’ claims concerning injuries during imaging examinations and took the patient’s perspective. Claims provide new information and therefore, it is important that each unit collects and analyses their own data. In this way, specific problems, such as delayed diagnoses or infections, can be highlighted. Radiology professionals are humans after all, and errors can never be completely avoided.

## Data Availability

The data that support the findings of this study are available from the Finnish Patient Insurance Centre but restrictions apply to the availability of these data, which were used under license for the current study, and so are not publicly available. Data are however available from the authors upon reasonable request and with permission of the Finnish Patient Insurance Centre.

## Abbreviations

HaiPro: Reporting System for Safety Incidents in Health Care Organizations
PVK: The Finnish Patient Insurance Centre (Potilasvakuutuskeskus in Finnish)
STUK: Radiation and Nuclear Safety Authority of Finland
VALVIRA: National Supervisory Authority for Welfare and Health
